# Impact of practice, provider and patient characteristics on delivering screening and brief advice for heavy drinking in primary healthcare: Secondary analyses of data from the ODHIN five-country cluster randomized factorial trial

**DOI:** 10.1080/13814788.2017.1374365

**Published:** 2017-10-12

**Authors:** Peter Anderson, Karolina Kłoda, Eileen Kaner, Jillian Reynolds, Preben Bendtsen, Myrna N. Pelgrum-Keurhorst, Lidia Segura, Marcin Wojnar, Artur Mierzecki, Paolo Deluca, Dorothy Newbury-Birch, Kathryn Parkinson, Katarzyna Okulicz-Kozaryn, Colin Drummond, Miranda G. H. Laurant, Antoni Gual

**Affiliations:** ^a^ Institute of Health and Society, Newcastle University Newcastle UK; ^b^ Department of Family Medicine, Maastricht University Maastricht the Netherlands; ^c^ Independent Laboratory of Family Physician Education, Pomeranian Medical University Szczecin Poland; ^d^ Psychiatry Dept. Neurosciences Institute, Hospital Clínic, IDIBAPS Barcelona Spain; ^e^ Department of Medical Specialist and Department of Medicine and Health, Linköping University Motala Sweden; ^f^ Radboud University Medical Center, Radboud Institute for Health Sciences, Scientific Center for Quality of Healthcare (IQ healthcare) Nijmegen the Netherlands; ^g^ Saxion University of Applied Sciences, Centre for Nursing Research Deventer/Enschede the Netherlands; ^h^ Program on Substance Abuse, Public Health Agency, Government of Catalonia Barcelona Spain; ^i^ Department of Psychiatry, Medical University of Warsaw Warsaw Poland; ^j^ National Addiction Centre, Institute of Psychiatry, King’s College London London UK; ^k^ Health and Social Care Institute, Teesside University Middlesbrough UK; ^l^ State Agency for Prevention of Alcohol-Related Problems Warsaw Poland; ^m^ National Institute for Health Research Biomedical Research Centre for Mental Health, South London and Maudsley NHS Foundation Trust London UK; ^n^ Faculty of Health and Social Studies, HAN University of Applied Sciences Nijmegen the Netherlands

**Keywords:** Primary healthcare, heavy drinking, screening and advice, training, patients, providers

## Abstract

**Background:** The implementation of primary healthcare-based screening and advice that is effective in reducing heavy drinking can be enhanced with training.

**Objectives:** Undertaking secondary analysis of the five-country ODHIN study, we test: the extent to which practice, provider and patient characteristics affect the likelihood of patients being screened and advised; the extent to which such characteristics moderate the impact of training in increasing screening and advice; and the extent to which training mitigates any differences due to such characteristics found at baseline.

**Methods:** A cluster randomized factorial trial involving 120 practices, 746 providers and 46 546 screened patients from Catalonia, England, the Netherlands, Poland, and Sweden. Practices were randomized to receive training or not to receive training. The primary outcome measures were the proportion of adult patients screened, and the proportion of screen-positive patients advised.

**Results:** Nurses tended to screen more patients than doctors (OR = 3.1; 95%CI: 1.9, 4.9). Screen-positive patients were more likely to be advised by doctors than by nurses (OR = 2.3; 95%CI: 1.4, 4.1), and more liable to be advised the higher their risk status (OR = 1.9; 95%CI: 1.3, 2.7). Training increased screening and advice giving, with its impact largely unrelated to practice, provider or patient characteristics. Training diminished the differences between doctors and nurses and between patients with low or high-risk status.

**Conclusions:** Training primary healthcare providers diminishes the negative impacts that some practice, provider and patient characteristics have on the likelihood of patients being screened and advised.

**Trial registration** ClinicalTrials.gov. Trial identifier: NCT01501552

KEY MESSAGES

- Training and support given to primary health care providers obviate to varying extents the detrimental impacts that patient and provider characteristics have in decreasing the likelihood of heavy drinking patients being screened and advised to reduce their drinking.

## Background

Within Europe, one-quarter of adults are heavy drinkers (women who consume 20 g + alcohol per day, and men 40 g + per day) [1]. While primary healthcare based screening and brief advice is effective in reducing heavy drinking [2], it is poorly implemented [3]. Previously, reporting the main results of the five-country ODHIN study (Catalonia, England, the Netherlands, Poland and Sweden), we found that training given to primary healthcare providers increased the proportion of consulting adult patients who were screened and advised for their heavy drinking [4].

In this paper, we report novel secondary analyses of the ODHIN data to explore: the extent to which practice, provider and patient characteristics affect the likelihood of patients being screened and advised; the extent to which such characteristics moderate the impact of training in increasing screening and advice; and the extent to which training mitigates any differences due to such features found at baseline. A priori, we expect patients’ gender and level of alcohol consumption to affect the proportion of patients screened and advised [5,6].

## Methods

Details of the trial protocol and the primary results have been published [4,7]. The focus of the study is changing provider behaviour through the interventions of training, financial reimbursement, and referral of screen-positive patients to web-based advice, singly or in combination. A factorial design was used, in which all allocation groups that received training, the focus of this study, were compared to those groups that did not. Practices with approximately 5000–20 000 registered patients were the unit of randomization and implementation. Practices were volunteers drawn from administrative or academic registries at national or regional levels until the required sample size of 120 (24 per country) was achieved. Eligible providers in each practice included any fully trained doctor, nurse or assistant, of whom 746 (36% of all eligible providers) took part in the study.

Practices were recruited during 2013. After formal agreement of the practice to take part, a four-week baseline measurement period occurred. After a two-to-six-week gap, the 12-week implementation intervention period occurred. Controls were given printed national guidelines on conducting screening and brief advice to reduce heavy drinking. Training consisted of two initial one-to-two hour’s face-to-face educational inputs and one (10–30 min) telephone support call during the 12-week implementation period. Using computerized randomization the ODHIN coordinating centre randomly allocated practices stratified by country. After the baseline measurement period, the practices and investigators were not blind to group allocation.

Practices were asked to screen all adult patients (aged 18 + years) who consulted the practice for whatever reason, using AUDIT-C [8]. Primary healthcare units (PHCU) have been invited to deliver brief advice to reduce heavy drinking of 5–15 min duration to screen-positive patients (AUDIT-C score 5 + in England and Catalonia, and 5 + (men) and 4 + (women) in Poland, the Netherlands and Sweden).

The two primary outcomes were the proportion of consulting adult patients screened, calculated in this paper at the level of the provider; and the proportion of screen-positive patients advised, calculated in this paper at the level of the patient. The distribution of the proportion screened per provider was highly positively skewed. For analyses, the proportion screened was dichotomized at the median of the whole provider sample (0.031). It was estimated that 120 practices (24 per country) would be needed for an 80% chance of detecting an increase in the proportion of consulting adult patients screened and screen-positive patients advised intervened from four to six per cent (ICC = 0.029) (alpha = 5%) [7]. One practice dropped out of the study after the baseline measurement. The analysis was conducted on an intention to treat basis.

Six practice variables were examined: practice type (health clinic or other); practice setting (exclusively urban or other); number of registered patients; number of providers per practice; ratio of doctors to non-doctors in the practice; and, screening method used (undertaken by doctors and nurses or by doctors alone). Four provider variables were examined: profession (nurses as opposed to doctor); gender; age and, number of adult consultations during the measurement period. Three patient variables were examined: gender; age; and AUDIT-C score, dichotomized to 8 + and less than 8 [9].

A generalized linear model was used employing a multi-level approach using country, PHCU, and provider, as appropriate, with random intercepts and slopes. Odds ratios (OR) are presented for dichotomous variables and regression coefficients (B) for continuous variables, both with 95% confidence intervals (95%CI). Analyses were conducted using IBM SPSS V23, procedure GENLIN.

## Results

The practice, participating provider and patient data are summarized in Table 1. Out of the 120 practices, data from 746 providers were analysed. During the four-week baseline measurement period, 9609 patient AUDIT-C record sheets were analysed, of which 1626 (16.9%) were screen positive. During the 12-week implementation period, 36 937 patient AUDIT-C record sheets were analysed, of which 5586 (15.1%) were screen positive.

**Table 1.** Descriptive data of the practice, provider and patient variables.

**Practice variables** (120 primary healthcare units analysed)	
• PHCU that are health clinics (%) as opposed to solo, duo or small group practices	63.7
• PHCU that are only urban (%) as opposed to rural or mixed urban/rural	48.1
• Mean (SD) registered population per PHCU	10 543 (4909)
• Mean (SD) number of providers per PHCU	17.4 (10.5)
• Mean (SD) ratio of doctors to non-doctors per PHCU	1.0 (0.76)
• Screening undertaken by doctors alone (% of PHCU), as opposed to both doctors and nurses	12.1
**Provider variables** (746 providers analysed)	
• Participating providers who are male (%) as opposed to female	24.7
• Participating providers who are doctors (%) as opposed to nurses	54.7
• Mean (SD) age (years) of participating providers	46.8 (9.3)
• Mean (SD) number of consulting adult patients per provider during measurement period	
Baseline	241 (186)
Implementation	651 (508)
• Total number of consultations during measurement period	
Baseline	179 954
Implementation	485 646
• Number (% of consulting patients) screened during measurement period	
Baseline	9609 (5.3)
Implementation	36 937 (7.6)
**Patient variables** (9609 patient records analysed at baseline; 36 937 analysed at implementation)	
• Mean (SD) age (years) of screened patients	
Baseline	57.7 (16.6)
Implementation	57.0 (17.2)
• Screened patients who are male (%)	
Baseline	49.8
Implementation	46.0
• Number (% of screened patients) AUDIT-C positive	
Baseline	1626 (16.9)
Implementation	5586 (15.1)
• Mean (SD) AUDIT-C score of screen-positive patients	
Baseline	6.54 (2.10)
Implementation	6.27 (1.94)
• Number (% screen-positive patients) advised	
Baseline	1202 (73.9)
Implementation	4866 (87.1)

PHCU: primary healthcare unit.

### Screening

During the four-week baseline period, of the six practice variables entered simultaneously in the model, only screening method was related to screening. The odds ratio (OR) for a provider being above the median of the proportion of consulting adult patients screened was 3.1 (95% CI: 1.5, 6.5) for providers whose practices undertook screening by both doctors and nurses as opposed to doctors alone. Of the four provider variables entered simultaneously in the model, only provider profession was related to screening. The odds ratio for a provider being above the median of the proportion of consulting adult patients screened was 3.1 (95%CI: 1.9, 4.9) for providers who were nurses as opposed to doctors.

Training was related to higher screening during the 12-week implementation period, with baseline screening, and the two other interventions (financial reimbursement and referral to web-based advice) added as covariates. The odds ratio for a provider being above the median of the proportion of consulting adult patients screened was 2.7 (95%CI: 2.1, 3.4) for providers who had received training, compared to those who had not. The impact of training was unaffected when the six practice variables were added to the model (OR = 2.8, 95%CI: 2.2, 3.6) or when the four provider variables were added to the model (OR = 2.6, 95%CI: 2.1, 3.5).

Training reversed the impact of practice screening method, but not the impact of the profession of the provider at baseline. In the presence of training during the 12-week implementation period, the odds ratio for a provider being above the median of the proportion of consulting adult patients screened was 4.3 (95%CI: 1.1, 16.9) for providers whose practices undertook screening by doctors alone as opposed to by both doctors and nurses.

### Advice giving

During the four-week baseline period, 1202/1626 screen-positive patients (73.9%) were given advice. Of the six practice variables entered simultaneously in the model, only screening method was related to advice giving. The odds ratio for a screen-positive patient being given advice was 3.8 (95%CI: 1.6, 8.8) for providers whose practices undertook screening by doctors alone as opposed to by both doctors and nurses. Of the four provider variables entered simultaneously in the model, only provider profession was related to advice giving. The odds ratio for a screen-positive patient being given advice was 2.3 (95%CI: 1.4, 4.1) for providers who were doctors as opposed to non-doctors. Of the three patient variables entered simultaneously in the model, only AUDIT-C score was positively related to advice giving. The odds ratio for a screen-positive patient being given advice was 1.9 (95%CI: 1.3, 2.7) for patients with an AUDIT-C score of 8 + compare to less than 8.

Training was related to higher advice giving during the 12-week implementation period, with the two other interventions (financial reimbursement and referral to web-based advice) added as covariates. The odds ratio for a screen-positive patient being given advice was 2.6 (95%CI: 2.1, 3.3) for providers who had received training, compared to those who had not. The impact of training was no longer significant when, of the six practice variables, screening method (but not any other practice variables) was added to the model. The impact of training was no longer significant when, of the four provider variables, the number of adult consultations (but not any other provider variables) was added to the model. The impact of training was not affected when adding the three patient variables to the model.

Training did not influence the impact of practice screening method on advice giving that was present at baseline but did affect the impact of the profession of the provider at baseline. In the presence of training during the 12-week implementation period, the odds ratio for a screen-positive patient being given advice was no longer significant (1.6, 95%CI: 0.8, 3.1) for providers who were doctors as opposed to nurses. Training did affect the impact of a screen-positive patient’s AUDIT-C score on advice giving that was present at baseline. Screen-positive patients with an AUDIT-C score of less than eight were more likely to be given advice in the presence of training than in the absence of training (OR = 1.7; 95%CI: 1.1, 2.8), whereas this was not the case for screen-positive patients with an AUDIT-C score of eight plus, who were equally likely to be given advice, independent of training (OR = 1.3; 95%CI: 0.8, 2.6).

## Discussion

### Main findings

During the four-week baseline period, we found that higher levels of screening were found in practices in which screening was undertaken by both nurses and doctors, rather than doctors alone and for providers who were nurses rather than doctors. We also found that screen-positive patients were more likely to be advised in practices in which screening was undertaken by doctors alone rather than by both nurses and doctors, by providers who were doctors rather than nurses, and in patients whose AUDIT- C score was eight plus, rather than less than eight.

We found that training led to higher screening and advice giving, with the impact of training largely unaffected by the practice, provider and patient variables that we analysed. Training tended to lessen the differences between nurses and doctors in screening and advice giving and mitigated the dichotomy in advice giving that was mediated by the AUDIT-C score.

### Strengths and weaknesses

A strength of the present study is the large number of practices, providers, and patients included, giving confidence in the findings across five different European countries. One weakness of the study is that the involved practices were volunteers. Previously, we indicated that providers from the volunteer practices seemed more motivated to work with drinkers than providers from the same country in general [4]. A second weakness of the present study is that the provider completed the tally sheet and an independent check was not done to prove the advice was carried out.

### Comparisons with existing literature

Two English studies found that the likelihood of screen-positive patients receiving advice to reduce their drinking was greater if patients were male, the higher the level of risky drinking, the longer the average consultation time, and if providers received training [5,6]. In this study, we found that patient’s gender did not influence the likelihood of screen-positive patients being advised, although the higher the level of risky drinking did. We did not measure consultation time but examined consultation rate, which had no independent effect on the proportion of patients screened or advised. The relationship with level of risky drinking was mitigated by training.

## Conclusions

At baseline, the only practice and provider characteristics that influenced screening and advice were professional roles. Nurses tended to screen more than doctors; doctors tended to advise screen-positive patients more than nurses. At baseline, screen-positive patients with higher risk were more likely to receive advice than those with lower risk. These differences due to practice, provider, and patient characteristics were largely mitigated by training.
